# Surgical ‘Safari’ *vs*. Educational Program: Experience with International Cardiac Surgery Missions in Nigeria

**DOI:** 10.21470/1678-9741-2020-0155

**Published:** 2020

**Authors:** Ikechukwu Andrew Nwafor, Aerra Vickram, Kennedy O. Osenmobor

**Affiliations:** 1 Department of Surgery, National Cardiothoracic Center of Excellence, University of Nigeria Teaching Hospital, Ituku-Ozalla, Enugu, Nigeria.; 2 Department of Surgery, Faculty of Medical Sciences, University of Nigeria, Ituku-Ozalla, Nsukka, Nigeria.; 3 Citizen Hospital, Hyderabad, Telangana, India.; 4 National Cardiothoracic Center of Excellence, University of Nigeria Teaching Hospital, Ituku-Ozalla, Enugu, Nigeria.

**Keywords:** Military Personnel, Leadership, Nigeria, Economic Development, Surgeons, Hospital, Teaching, Cardiac Surgical Procedures, Growth and Development

## Abstract

**Introduction:**

In any country, the development and growth of open-heart surgery parallel stable political climate, economic growth, good leadership, and prudent fiscal management. These were lacking in Nigeria, which was under a military rule. The enthronement of democratic rule, in 1999, has caused desirable changes. The objective of this study is to report our experience with foreign cardiac teams that visited the National Cardiothoracic Center of Excellence, University of Nigeria Teaching Hospital, for seven years, in order to restart its open-heart surgery program.

**Methods:**

To achieve the desired open-heart surgery training, our center received regular and frequent visits from foreign cardiac teams who would perform open-heart surgery with the local team.

**Results:**

During the period of seven years, a total of 266 open-heart operations involving both adults and children were performed, with a mean of 38 cases per year; 150 (54.4%) males and 116 (43.6%) females were treated, with a ratio of 1.0:0.8. Six different teams visited the center at different periods.

**Conclusion:**

After these years of cardiac missions to our center, the experience of the local team, especially the surgeons, is far from desirable because each team visit usually lasted about a week or two and each team, with exception of the CardioStart International/William Novick Global Cardiac Alliance, adopted the surgical ‘safari’ method.

**Table:** 

Abbreviations, acronyms & symbols				
AML	= Anterior mitral valve leaflet		PAVD	= Partial anomalous pulmonary venous drainage
ASD	= Atrial septal defect		PDA	= Patent ductus arteriosus
AVCD	= Atrioventricular canal defect		PS	= Pulmonary stenosis
AVS	= Aortic valve stenosis		RV	= Right ventricle
CHD	= Congenital heart disease		SAHN	= Save-A-Heart Nigeria
ECG	= Electrocardiogram		SAM	= Subaortic membrane
ICU	= Intensive care unit		TA	= Truncus arteriosus
LA	= Left atrium		TETF	= Tertiary Education Trust Fund
LSVC	= Left superior vena cava		TOF	= Tetralogy of Fallot
MAPCAS	= Major aortic pulmonary artery collaterals		TVR	= Tricuspid valve regurgitation
MVR	= Mitral valve regurgitation		UNEC	= University of Nigeria, Enugu *campus*
NCTCE	= National Cardiothoracic Center of Excellence		UNTH	= University of Nigeria Teaching Hospital
NGOs	= Nongovernmental organizations		VOOM	= Vincent Ohaju Memorial
OHS	= Open-heart surgery		VSD	= Ventricular septal defect
PA	= Pulmonary artery			

## INTRODUCTION

The University of Nigeria Teaching Hospital (UNTH), in Ituku-Ozalla, Enugu, is the medical wing of the University of Nigeria Nsukka, or UNN, and it was established in 1970^[1]^. Four years later, in 1974, it became the first hospital in the country to carry out the first open-heart surgery (OHS) in Nigeria, with a foreign cardiac team led by a British-Egyptian, Sir Dr Yacoub, and an indigenous team led by late professor Fabian Udekwu^[2]^. As a result, the Federal Government of Nigeria, in 1978, designated UNTH, Enugu, a National Cardiothoracic Center of Excellence (NCTCE)^[3]^.

Since that time, the center has been making efforts to establish itself as a leader in OHS, not only in Nigeria, but also in West Africa subregion^[4]^. However, the military rule coupled with its near total neglect of health system in the country led to the collapse of the center due to brain drain and inadequate facilities.

With the enthronement of democracy in Nigeria in 1999^[5]^, efforts have been made to restart the open-heart program in the center. The options considered were staff training and equipment procurement. It was considered that the best way to achieve the desired training in an emerging country like ours was by receiving regular visits from foreign cardiac teams in the center who would perform OHS alongside the local team (cardiac mission model)^[6,7]^. This model has the advantage of treating the country’s patients at the same time. In addition, the model makes it possible for the visiting team to work in the usual environment of the local team. Moreover, the model makes for the improvement in the hospital’s infrastructure and equipment. Ideally, it ought to provide education, training, and sufficient experiences which eventually lead to full functional independent cardiac services that are sustainable over time^[8]^.

Other options included sending the local team to established centers (*e.g*., India) for hands-on-training for a period not less than two years^[9]^. Furthermore, members of the local team may undergo abroad training on his or her own personal arrangement at different times and in different established centers. Training a complete cardiac team from a developing country is cumbersome, complex, and expensive^[10]^. In addition, to train a foreign resident in that setting is an added challenge^[11]^.

The cardiac mission model, however, is not sustainable, as a lot of effort and expenditure are allocated towards surgery in few patients, led by experts who can afford short periods of stay away from their regular jobs^[12]^. Huge expenses are also incurred emanating from the air tickets of the team, procurement of visas, freight of needed equipment, and consumables including customs or excise duties at the airport or wharf. Accommodation and security of the visiting team are also an additional cost. Some foreign teams charge exorbitant fees, which are not affordable, and efforts are made to avoid high-risk cases, which would consume scarce resources, operating time, and tie down intensive care unit beds^[13]^.

Efforts to establish a functional OHS program in Nigeria at other centers - like Lagos State University Teaching Hospital, or LASUTH, Ikeja, University College Hospital, or UCH, Ibadan, Lagos University Teaching Hospital, or LUTH, Idi-Araba, Gwagwalada Teaching Hospital, or GTH, Abuja, Obafemi Awolowo University Teaching Hospital, or OAUTH, Ile-If, Dr Joseph Nwiloh Cardiac Hospital, Adazi-ala, Anambra state, Ahmadu Bello University Teaching Hospital, or ABUTH, Zaria, and Orthan Dan Fodio University Teaching, or ODUTH, Sokoto state - have abysmally failed over the years because of emphasis on the surgical ‘safari’ method rather than on an educational program^[9]^.

With return to democratic rule in 1999, efforts were made to restart the open-heart program at NCTCE, UNTH, Enugu. A purpose building, which was started in 1986, was completed and commissioned in 2001. The OHS program was going on. Unfortunately, the Enugu location had to be abandoned during moving to Ituku-Ozalla center, in 2007, which by then had no befitting arrangement for OHS. The Ituku-Ozalla center became ready for OHS in 2013.

## METHODS

Different cardiac mission teams visited and treated patients for about 7.5 years (March, 2003 to October, 2019). A retrospective study was then carried out to compare surgical safari and educational program with a view to determine the levels of participation and competence of the local team, especially the surgeons. The data obtained from the hospital records database were the patients’ demographics, the number and type of cases managed, the number and frequency of visits of different cardiac mission teams, the level of participation and competence of a multidisciplinary local team, and funding of the program. The number of patients that had extracardiac surgery performed by the local and or foreign teams was excluded from the study.

Data were analyzed using IBM Corp. Released 2011, IBM SPSS Statistics for Windows, Version 20.0, Armonk, NY: IBM Corp. Rates and proportions were calculated with 95% confidence interval. The proportions were compared using Student’s t-tests. Level of significance was set at < 0.5.

### Ethical Approval

This research work is an audit of our work at NCTCE and is exempted from ethical approval.

## RESULTS

During the study period, a total of 266 cardiac surgeries in 242 patients, involving both adults and children, were performed with a mean of 38 cases per year.

Table 1 displays the seven years of foreign cardiac mission in Nigeria with the number of patients; 33 mission trips were recorded, and 242 patients were treated.

**Table 1:** Seven years of foreign cardiac missions at the National Cardiothoracic Center of Excellence, University of Nigeria Teaching Hospital, Enugu, Nigeria.

S/No.	Year	No. of missions	No. of patients treated	Percentages (%)
1	2013	4	24	9.9
2	2014	3	17	7.0
3	2015	5	42	17.3
4	2016	5	53	21.9
5	2017	6	44	18.2
6	2018	5	40	16.5
7	2019	5	22	9.1
**Total**		**33**	**242**	**100**

Table 2 shows the distribution of the patients’ age ranges, including gender. The most affected were the age ranges of 1120 and 0-10 years. There were 112 females and 130 males.

**Table 2:** Age ranges and gender distribution of patients treated by the visiting cardiac teams.

S/No.	Age ranges (years)	Females	Males	Total	Percentages (%)
1	0-10	25	30	55	22.7
2	11-20	28	30	58	23.9
3	21-30	10	9	19	7.9
4	31-40	14	12	26	10.7
5	41-50	11	16	27	11.2
6	51-60	11	13	24	9.9
7	61-70	9	12	21	8.7
8	71-80	2	5	7	2.9
9	81-90	2	3	5	2.1
**Total**	** **	**112 (46.3%)**	**130 (53.7%)**	**242**	**100**

Table 3 shows the distribution of adult cases managed. Heart valve surgery dominated, accounting for 108 (71.5%) cases, followed by coronary artery disease. A total of 151 cases were treated.

**Table 3:** Types and number of adult cases treated.

S/No.	Types of adult cardiac cases	Number	Percentages (%)
1	Heart valves	108	71.5
2	Cardiac tumors (atrial myxoma)	4	2.6
3	Adult CHD	20	17.4
4	Coronary artery disease	11	7.3
5	Vegetations on the tricuspid	1	0.7
6	Endocarditis	1	0.7
7	Ascending aortic aneurysms	6	3.9
**Total**	** **	**151**	**100**

CHD=congenital heart disease

**Fig. 1:**
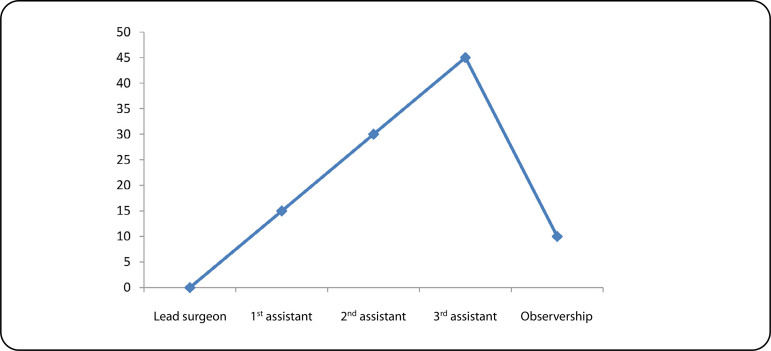
Level of participation of local surgeons.

Figure 1 shows the level of participation of local surgeons. Throughout the period of seven years, no local surgeon ever led an OHS case. This partly accounts for the failure of the program because there was no sustainability.

**Fig. 2:**
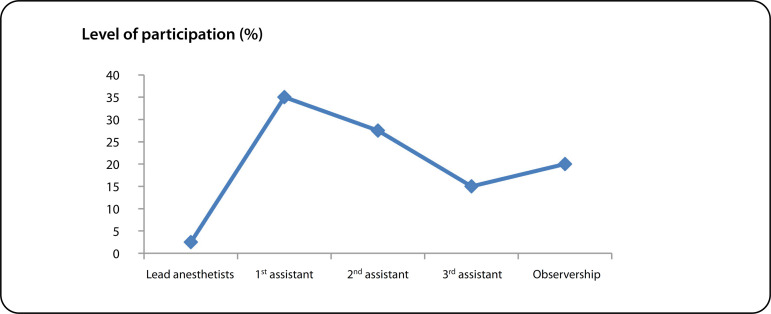
Level of participation of local anesthetists.

Figure 2 shows the level of participation of local anesthetists. Throughout the period of seven years, less than 5% of local anesthetists led an OHS case. This partly accounts for the failure of the program because there was no sustainability.

**Fig. 3:**
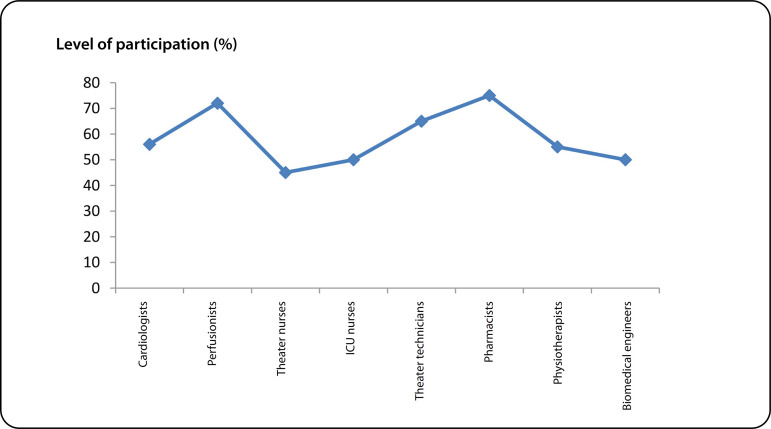
Level of participation of other multidisciplinary cardiac team members. ICU=intensive care unit

Figure 3 shows the level of participation of other members of local multidisciplinary cardiac team. Their participation was relatively better than those from anesthetists and surgeons. According to the hierarchy of training, there was no sustainability.

Table 4 shows the distribution of congenital heart diseases (CHD), both simple and complex types, treated. They are divided into isolated and combined lesions. Isolated tetralogy of Fallot (TOF) and ventricular septal defect (VSD) were dominant, representing 27.8% and 24.3% of the cases, respectively.

**Table 4:** Types and number of pediatric cases treated.

S/No.	Types of pediatric cases treated	Number	Percentages (%)
1	TOF	32	27.8
2	VSD	28	24.3
3	ASD	9	7.8
4	MVR (rheumatic)	2	1.7
5	VSD +ASD	3	2.6
6	VSD + PDA	11	9.5
7	ASD + PAVD	3	2.6
8	PA + MAPCAS	1	0.9
9	AVCD	5	4.3
10	TA, type 1	3	2.3
11	Severe TVR	2	1.7
12	VSD + SAM	2	1.7
13	Severe MVR + TVR + dilated LA	1	0.9
14	ASD + Cor-triatriatum	1	0.9
15	VSD + ASD + cor-triatriatum	1	0.9
16	VSD + ASD + AML	1	0.9
17	Common atrium + PDA	1	0.9
18	Large ASD + cor-triatriatum + valvular & supravalvular PS	1	0.9
19	VSD + ASD + SAM	1	0.9
20	ASD + PS	2	1.7
21	Severe PS + TVR + RV thrombus	1	0.9
22	SAM (AVS) + PDA	3	2.6
23	ASD + PS + LSVC	1	0.9
**Total**	** **	**115**	**100**

AML=anterior mitral valve leaflet; ASD=atrial septal defect; AVCD=atrioventricular canal defect; AVS=aortic valve stenosis; LA=left atrium; LSVC=left superior vena cava; MAPCAS=major aortic pulmonary artery collaterals; MVR=mitral valve regurgitation; PA=pulmonary artery; PAVD=partial anomalous pulmonary venous drainage; PDA=patent ductus arteriosus; PS=pulmonary stenosis; RV=right ventricle; SAM=subaortic membrane; TA=truncus arteriosus; TOF=tetralogy of Fallot; TVR=tricuspid valve regurgitation; VSD=ventricular septal defect

**Fig. 4:**
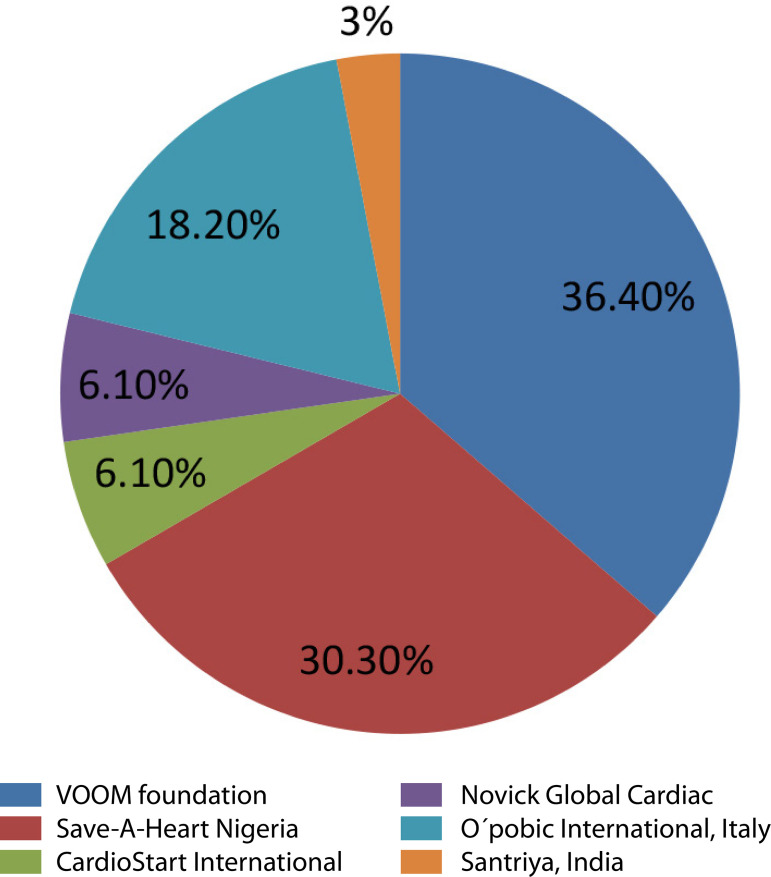
Different visiting foreign cardiac teams and number of missions (%). VOOM=Vincent Ohaju Memoria

Figure 4 shows the distribution of the foreign cardiac teams that visited and operated on patients under mission model. Here the two nongovernmental organizations (NGOs) (Vincent Ohaju Memorial [VOOM] and Save-A-Heart Nigeria [SAHN]), both led by Nigerians in Diaspora, dominated. Both emphasized on surgical safari and this accounted for the failure of the program after seven years.

Tables 5 and 6 shows the distribution of sources of fund for the OHS program and methods of fund utilization. Here, the Tertiary Education Trust Fund (TETF) constituted the major share. However, after seven years of mission model, most of the equipment have spoilt without replacement. This also accounts for why the program is not sustainable. Closely following TETF from the Federal Government of Nigeria through the National Universities Commission, or NUC, are the Federal Ministry of Health and the UNTH management for their varied roles, as displayed in the tables.

**Table 5:** Funding of the open-heart surgery program at the National Cardiothoracic Center of Excellence, University of Nigeria Teaching Hospital (UNTH), Enugu.

S/No.	Sources of fund	Description of fund utilization	Remarks
1	Tertiary Education Trust Fund	Medical equipment	40% (see Table 6)
2	Federal Ministry of Health	Payment of salaries of local multidisciplinary cardiac team	20%
3	UNTH management	Air tickets, visas, accommodation, transportation, feeding and security of visitors, consumables, generator (power supply), excise duties of donated equipment	20%
4	Foreign donations (foreign missions)	Used equipment, consumables, honorarium, air tickets	8%
5	Rotary Club	Payment of surgical fees	2%
6	Patients and relatives	Payment of surgical fees, blood donations	2%
7	Bigaard Seminary School Enugu (seminarians), medical students (UNEC)	Blood donations	1.5%
8	Public-spirited individuals	Payment of surgical fees and blood donations	1.5%
9	Enugu state government	Payment of surgery fees for pediatric cases	5%

UNEC=University of Nigeria, Enugu campus

## DISCUSSION

OHS is a type of surgery that requires an exposure of the internal structure of the heart. This often requires cardiopulmonary bypass. Due to the functions of the heart, this procedure requires highly skilled manpower, sophisticated equipment, and infrastructure^[14]^. These essentials are lacking in many countries in sub-Saharan African countries, except South Africa, Egypt, and Sudan. In many West African countries, heart surgery started with closed heart procedure (mitral commissurotomy, repair of patent ductus arteriosus [PDA], pericardiectomy, etc) by trained cardiothoracic surgeons from the United Kingdom, United States of America, and France, as early as 1960^[13]^. However, countries like Ghana, Nigeria, and Côte d’Ivoire began to perform OHS with aid of foreign cardiac teams^[15]^. This momentum was sustained through the 1980s before most of the programs failed due to economic challenge, occasioned by military rule and civil strife, including interprofessional conflicts^[16]^.

In Nigeria, a foreign cardiac team with the local multidisciplinary cardiac team performed the first OHS in our institution. This singular effort, in addition to many others, endeared the institution to the Federal Government of Nigeria. Consequently, the local personnel and equipment, including the infrastructure that carried the first OHS, were by executive order of the Federal Government of Nigeria proclaimed a NCTCE in 1984^[3]^. Shortly afterwards, the center’s activities dwindled due to military rule, corruption, and brain drain. Between 1974 and 2000, about 102 cases of OHS were carried out^[6]^.

With return to democratic rule in 1999, efforts were made to revitalize the center by continuation of the foreign cardiac mission model, using Nigerians in Diaspora as anchors. The first mission in a democratic set-up was by the International Heart Foundation, or ICF, in partnership with Kanu Heart Foundation, or KHF, precisely in 2003^[17]^. Ten years later, other cardiac missions started and became relatively regular (Table 1 and Figure 4).

During these periods, about 266 cases have been treated over seven years, averaging 38 cases per year; 130 (53.7%) males and 112 (46.3%) females, both adults and children, were involved (Table 2). Male to female ratio was 1.0:0.9. In these series, heart valve diseases (71.5%), followed by adult CHD (17.4%), were the commonest indications for intracardiac surgery in adult patients (Table 3). In the pediatric group, isolated TOF (n=32, 27.8%) and isolated VSD (n=28, 24.3%) followed by VSD and PDA (n=11, 9.5%) were the commonest indications for OHS (Table 4). For the first time, complex types of CHD were successfully managed^[18]^. They were truncus arteriosus type 1^[19]^ and congenital tricuspid valve disease (congenital absence of both anterior and posterior leaflets), as well as pulmonary atresia with major aortic pulmonary artery collaterals (Table 4). Greater percentages of patients with mitral and aortic valve diseases had prosthetic replacement in view of the fact that they were predominantly rheumatic and also that the chances of having a second surgery would be difficult financially.

The age of the patients operated on ranged from five months to 86 years, with a mean of 43.2 years. The age range of patients mostly involved in the OHS procedure was 11-20 years (n=58, 23.9%), followed by 0-10 years (n=55, 22.7%). The least affected age ranges were 71-80 and 81-90 years (n=7, 2.9% and n=5, 2.1%, respectively) (Table 2).

Despite the reasonable number of cases performed, the participation of the local surgeons and anesthetists was very low (Figures 1 and 2). These are the highest skilled professionals in the hierarchy of a cardiac team based on their training. In this model, that lasted a week or two (each visit) with irregular frequency, training of the surgeons or the anesthetists was highly inadequate. The different foreign surgeons were often uncomfortable allowing a local surgeon to lead an OHS case. Also, some Nigerians in Diaspora running the NGOs (VOOM and SAHN) saw the mission model as a surgical safari rather than an educational program (Figure 3). To this end, they did not see the long-term benefit of a local team taking over the performance of the OHS program.

Therefore, emphasis was that a cardiac surgeon and or anesthetist should undergo proper hands-on-training in reputable centers abroad with high volume of surgeries in a year^[9]^. The European Association of Cardiothoracic Surgeons and its International Committee do not believe that the best solution in achieving a sustainable cardiac program in a developing country is by sending a foreign cardiac team there^[20]^. However, in a similar study by Velebit et al.^[7]^, a team of fully trained specialists did a cardiac mission in Tbilisi, Georgia, for a period of five years where the frequency was monthly, with one-week duration, and with an average of 10 times in a year. After five years, the local team was able to become independent and had performed 181 cases out of 204, within the first seven months in 2008, while the remaining was done by a foreign team during irregular support visits related to difficult cases^[7]^.

In Nigeria, with a population of 200 million people^[21]^ and with supposedly six government centers, ours inclusive, trying to perform an OHS through mission model was far from ideal. The ideal would have been the government supporting one or two centers to become proficient. It is these proficient centers that ultimately will drive the local growth of skills in OHS nationally. Other middle skilled members of the local team were easily taught, and their level of participation was high (Figure 3).

From the onset, three modalities were considered in revitalizing the OHS program in our center. The cheapest of them was the mission model. In low-income countries like Nigeria, with double burden of diseases (communicable and noncommunicable), getting a near ideal cardiac center requires huge investments in procuring high-tech equipment, building infrastructure, and training personnel. Indeed, it would not have been possible without aids from the Nigerian government, Nigerians in Diaspora, public-spirited individuals, and foreign donations (Table 5). Most of the countries in the sub-region are very poor and in a study by Edwin et al.^[22]^, it was shown that no existing cardiac center in the sub-region came into being without government interventions^[23]^. Thus, as pointed out by Dearani et al.^[24]^, humanitarian outreach activities should focus on education and sustainability and surgical tourism should be limited to those centers that will never have the capacity to have a free-standing cardiothoracic program^[22]^. Evidently, it was documented that success of cardiac mission should not be measured by the number of successful operations of any given mission, but by the successful operations the local team were able to perform after the departure of the visitors^[24,25]^. In our own situation, we had 33 mission trips and carried out about 266 cardiac procedures in 242 patients over a seven-year period, yet the locals had not transited to independence even for minor cases like atrial septal defect, VSD, and minor forms of TOF, as well as valves in adults (Table 1). The main goal of the mission should be to provide teaching to local staff and implement methods and techniques to support the improvements in the care of patients in the long run^[22]^.

## CONCLUSION

The adoption of a cardiac mission model to revitalize the OHS program following the enthronement of democratic rule in our country as well as helping indigent patients with both congenital and acquired heart diseases and creating awareness was good. But this surgical safari was not sustainable. However, this method is like giving someone a fish any time he demanded it. The best way was to have incorporated teaching the person how to fish, *i.e*., equipping and developing the local team for sustainability.

**Table:** 

**Authors' roles & responsibilities**	
IAN	Substantial contributions to the design of the work; or the acquisition, analysis, or interpretation of data for the work; drafting the work or revising it critically for important intellectual content; agreement to be accountable for all aspects of the work in ensuring that questions related to the accuracy or integrity of any part of the work are appropriately investigated and resolved; final approval of the version to be published
AV	Drafting the work or revising it critically for important intellectual content
KOO	Substantial contributions to the design of the work; or the acquisition, analysis, or interpretation of data for the work; agreement to be accountable for all aspects of the work in ensuring that questions related to the accuracy or integrity of any part of the work are appropriately investigated and resolved; final approval of the version to be published
